# Current Status of Monocyte Differentiation-Inducing (MDI) Factors Derived from Human Fetal Membrane Chorion Cells Undergoing Apoptosis after Influenza Virus Infection

**DOI:** 10.4137/grsb.s374

**Published:** 2007-11-12

**Authors:** Noboru Uchide, Hiroo Toyoda

**Affiliations:** 1 Department of Clinical Molecular Genetics, School of Pharmacy, Tokyo University of Pharmacy and Life Sciences, 1432-1 Horinouchi, Hachioji, Tokyo 192-0392, Japan; 2 Department of Medicine, Division of Infectious Disease, University of California, San Francisco, 513 Parnassus Avenue, San Francisco, CA 94143, U.S.A

**Keywords:** Fetal membranes, influenza virus, apoptosis, macrophages, phagocytosis, cytokines

## Abstract

Influenza virus infection induces apoptosis and the expression of a set of pro-inflammatory cytokine genes, such as interleukin (IL)-6, tumor necrosis factor (TNF)-α, interferon (IFN)-β and IFN-γ, in cultured human fetal membrane chorion cells. Monocyte differentiation-inducing (MDI) activity in culture supernatants is simultaneously increased by the virus infection. The MDI activity is predominantly influenced by IL-6 molecule in culture supernatants, and partly by TNF-α and IFN-β, but not IFN-γ, molecules. The MDI factors are able to induce the mRNA expression of macrophage class A scavenger receptor (SR-A), which is one of adhesion and apoptotic cell-recognizing molecules, and gp91^phox^, which is a catalytic subunit of reduced nicotinamide adenine dinucleotide phosphate (NADPH) oxidase enzyme complex, on monocytic cells. As a result, monocytes are initiated to differentiate into well-matured macrophages capable of adhering and producing superoxide through NADPH oxidase. The matured macrophages, obtained from human monocytic leukemia THP-1 cells by the treatment with MDI factors, phagocytose apoptotic chorion cell debris resulting from the virus infection. Subsequent to phagocytosis, an abrupt increase of superoxide production by macrophages may occur. In this article, we summarize recent knowledge about the MDI factors derived from human fetal membrane chorion cells undergoing apoptosis after influenza virus infection, and discuss their possible pathological roles during pregnancy.

## Introduction

The past influenza pandemics clearly demonstrate that influenza virus infection during pregnancy has been implicated as one of the causes of premature delivery, abortion and stillbirth (Hardy et al. 1961; Harris, 1919). Influenza virus type A infection during pregnancy is still an etiological awareness at the present day (Stanwell-Smith et al. 1994), and many pregnant women (0.25%–0.51%) are hospitalized for the influenza infection (Hartert et al. 2003; Neuzil et al. 1998). Influenza A viruses have been isolated from placenta and amniotic fluid during the third trimester in fatal (Jewett, 1974; Yawn et al. 1971) and non-fatal cases (McGregor et al. 1984). The occurrence of viremia with influenza virus has been substantiated (Lehmann and Gust, 1971; Naficy, 1963; Ritova et al. 1979; Stanley and Jackson, 1966), and it has been demonstrated that influenza viruses spread to placenta, fetal membranes and amniotic fluid via the bloodstream (Rushton et al. 1983; Sweet et al. 1977). Therefore, it is important to study the virulence of influenza virus infection to gestational tissues in order to understand the etiology of influenza-associated interruption of pregnancy.

Human fetal membranes are composed of amnion, chorion and decidua tissues (Lavery, 1987). They form boundaries between the fetus and the external world, playing a critical role as defensive barriers against infection in order to maintain normal pregnancy (Grossman and Dennis, 1987). To elucidate the virulence of influenza virus infection to human fetal membranes, we have been investigating the induction of apoptosis and pro-inflammatory cytokine gene expression in primary cultured chorion and amnion cells. We reported that influenza virus replicates in both cultured chorion and amnion cells, while in only chorion cells the virus infection induces apoptosis and the expression of a set of pro-inflammatory cytokine genes, such as interleukin (IL)-1β, IL-6, tumor necrosis factor (TNF)-α, interferon (IFN)-β and IFN-γ (Uchide et al. 2002a, 2002b). It should be noted that these phenomena are not observed in cultured amnion cells, yet the virus replicates in the cells (Uchide et al. 2002a, 2002b). On the basis of these results, we have suggested that fetal membrane chorion cells play a pivotal role in the pathogenesis of pregnancy-associated complications during intrauterine influenza virus infection through the induction of apoptosis and pro-inflammatory cytokine gene expression (Uchide et al. 2005a).

Apoptosis induction has been defined as the elimination of dying cells without inducing an inflammatory response (Wyllie et al. 1980). However, this conventional definition may not be fit in a certain situation, such as pathogen invasion, that induces an inflammatory response, resulting in the activation of an immune response (Restifo, 2000). Recent studies suggest that the phagocytosis of influenza virus-infected cells undergoing apoptosis by macrophages plays a critical role in the presentation of viral antigen to T lymphocytes, the inhibition of virus growth and the prevention of virus dissemination in the infected organs (Albert et al. 1998; Fujimoto et al. 2000; Mori et al. 2002; Shiratsuchi et al. 2000; Watanabe et al. 2002, 2004, 2005). It has been postulated that immature monocytes in the bloodstream are able to differentiate to macrophages in order to phagocytose apoptotic cells resulting from the virus infection. However, it is still not clear how monocytes are attracted to virus-infected cells and then differentiate to macrophages.

Interestingly, we found that monocyte differentiation-inducing (MDI) activity in the culture supernatants of chorion cells is increased by influenza virus infection in the process of apoptosis (Uchide et al. 2002c). In this article, we summarize recent knowledge about the MDI factors derived from human fetal membrane chorion cells undergoing apoptosis after influenza virus infection, and discuss their possible pathological roles during pregnancy.

## Molecular and Biological Characteristics of MDI Factors

### Monocyte differentiation

Human monoblastic leukemia THP-1 cells are shown to differentiate to macrophages capable of adhering to a substrate and phagocytosing yeasts and immunoglobulin G-coated sheep red blood cells after the treatment with 12-*O*-tetradec-anoylphorbol 13-acetate (TPA) (Tsuchiya et al. 1982). We examined the effect of heated culture supernatants of chorion and amnion cells on monocyte differentiation using THP-1 cells (Uchide et al. 2002c). THP-1 cells became adherent to plastic plates by the incubation with heated culture supernatants of influenza virus-infected chorion cells (IV-C-sup), the extent of which was much higher than that with culture supernatants of mock-infected chorion cells (Mock-C-sup). Interestingly, THP-1 cells did not acquire the adherence activity when the cells were incubated with culture supernatants of mock and influenza virus-infected amnion cells (Mock-A-sup and IV-A-sup, respectively). The Giemsa staining method showed that non-treated THP-1 cells were round, the nucleocytoplasmic ratio was >1, and the cytoplasm was highly basophilic with a few vacuoles. In contrast, the THP-1 cells adhered to coverslips after the incubation with IV-C-sup were irregularly shaped, the nucleocytoplasmic ratio decreased to <1, and the cytoplasm was weakly basophilic with many vacuoles. Furthermore, adhered THP-1 cells phagocytosed many fluorescent latex particles. These results demonstrate that THP-1 cells are morphologically and functionally differentiated to macrophages by the incubation with heat-stable soluble factors in IV-C-sup. Therefore, we have suggested for the first time that influenza virus-infected chorion cells undergoing apoptosis secrete heat-stable MDI factors (Uchide et al. 2002c).

### Phagocytosis of apoptotic cell debris

We investigated the effect of soluble factors, which are derived from chorion cells, on phagocytotic reactions by macrophages (Uchide and Toyoda, 2007b). Since chorion cells were detached from a substrate due to apoptosis resulting from the virus infection (Uchide et al. 2002a), the apoptotic chorion cell debris was collected for the analysis. Adherent THP-1 cells were obtained by the treatment with IV-C-sup and then incubated with the apoptotic chorion cell debris in IV-C-sup or fresh medium. When incubated with IV-C-sup viral nucleoprotein-positive particles were detected within adherent THP-1 cells by immunohistochemical analysis, while such particles were not detected when incubated with fresh medium. These results suggest that the matured macrophages, obtained by the treatment with MDI factors, phagocytose apoptotic chorion cell debris resulting from the virus infection, and moreover that chorion cells secrete heat-stable soluble factors to support phagocytotic reaction by macrophages.

To elucidate the presence of heat-stable soluble factors to support phagocytosis by macrophages, we examined the effect of fresh medium, heated Mock-C-sup, heated IV-C-sup and non-heated IV-C-sup on adhesion and phagocytosis activities of macrophages obtained by the treatment with MDI factors (Uchide and Toyoda, 2007b). When adherent THP-1 cells obtained after the treatment with IV-C-sup were incubated with apoptotic chorion cell debris in fresh medium, the total cell number of adherent THP-1 cells was significantly decreased as compared to IV-C-sup, and no phagocytosing THP-1 cells were observed as described above. In contrast to fresh medium, the incubation in Mock-C-sup retained the adhesion and phagocytosis activities as well as IV-C-sup. The incubation in non- heated IV-C-sup somewhat increased the total cell number of adhered THP-1 cells as compared to IV-C-sup, accompanying by the increase in number of phagocytosing THP-1 cells. These results substantiated that chorion cells secrete heat-stable soluble factors with the activity of maintaining both adhesion and phagocytosis activities of macrophages irrespective of influenza virus infection. It is known that macrophage migration inhibitory factor (MIF) maintains macrophage adhesion and is heat-stable (Weiss and Glaves, 1975), and moreover that MIF stimulates the phagocytotic activity of macrophage-like RAW 264.7 cells (Onodera et al. 1997). Additionally, MIF mRNA and its protein are detected in amniochorion tissues (Marvin et al. 2002; Ietta et al. 2002). Therefore, it seems that the chorion cell-derived MIF-like activity plays a fundamental role in the functions of matured macrophages, such as adhesion and phagocytosis. Recently, Hashimoto and co-workers reported that the phagocytotic activity of macrophages is stimulated in vitro by heat-labile substances released from influenza virus-infected HeLa cells undergoing apoptosis (Hashimoto et al. 2007), the molecules of which seem to be different from heat-stable factors with MIF-like activity in IV-C-sup according to their physicochemical properties.

### Superoxide production

The cellular biological characteristics of MDI factors were further analyzed by the nitroblue tetrazolium (NBT) reduction test for measuring the ability of superoxide production (Uchide et al. 2006b). When human peripheral blood monocytes as well as monoblastic THP-1 and histiocytic U937 leukemia cells were treated with IV-C-sup, these cells acquired the ability of NBT reduction, which was much higher than that with Mock-C-sup. The induced NBT reduction was inhibited by the addition of superoxide dismutase and diphenyleneiodonium chloride, an inhibitor for reduced nicotinamide adenine dinucleotide phosphate (NADPH) oxidase, indicating that NBT was reduced by superoxide resulting from the activation of NADPH oxidase. In contrast, the treatments with Mock-A-sup and IV-A-sup had no effect on the NBT reduction ability in THP-1 cells. Therefore, these results suggest that the MDI factors induce the differentiation of human peripheral blood monocytes and monocytic lineage cells to well-matured macrophages capable of producing superoxide through NADPH oxidase.

### Gene regulation by MDI factors

Our unpublished data showed that a large proportion (76%) of THP-1 cells acquired both adherence and superoxide production abilities after the incubation with IV-C-sup, but a small proportion (24%) acquired only superoxide production ability. It is well known that class A scavenger receptor (SR-A) on the cell surface of macrophages is one of adhesion and apoptotic cell-recognizing molecules (Peiser and Gordon, 2001), and the treatment with TPA dramatically induces the expression of SR-A mRNA in THP-1 cells (Liao et al. 1999). Additionally, membrane-integrated protein gp91^phox^, existing as a heterodimer with p22^phox^, functions as the catalytic core of the phagocyte NADPH oxidase (Minakami and Sumimoto, 2006), and the treatment with IFN-γ or TPA induces the expression of gp91^phox^, not p22^phox^, mRNA in THP-1 cells (Newburger et al. 1988). We investigated the effect of MDI factors on SR-A, gp91^phox^ and p22^phox^ mRNA expression in adhered and suspended THP-1 cells discriminately (Uchide et al. 2002c; our unpublished data). The levels of SR-A mRNA expression were increased in only adhered, not suspended, THP-1 cells after the incubation with IV-C-sup, while the levels of gp91^phox^ mRNA expression were increased in both adhered and suspended THP-1 cells. However, the levels of p22^phox^ mRNA expression were not changed. These results suggest that MDI factors induce the expression of SR-A and gp91^phox^ genes, resulting in the differentiation of monocytes to well-matured macrophages capable of adhering and producing superoxide by NADPH oxidase.

### Possible member of MDI factors

Physicochemical and biochemical properties of the MDI factors in IV-C-sup were investigated (our unpublished data). The MDI activity was remained in the inner dialysate of IV-C-sup and stable at 56 °C but labile at 100 °C. Treatments of IV-C-sup with trichloroacetic acid and trypsin entirely decreased the MDI activity. In contrast, the treatment with dithiothreitol significantly increased the activity. These results suggest that the MDI factors in IV-C-sup are heat-stable peptidyl macromolecules, and a reduced form of the factors is more active than its oxidized form.

Physiological program of monocyte differentiation to macrophage normally proceeds under the control of several cytokines in a coordinate manner. For example, IL-6 induces the differentiation of human monocytic leukemia cell lines including THP-1 cells to macrophages capable of producing superoxide, the activity of which is synergistically enhanced in combination with either IL-1, TNF-α or IFN-γ (Noda et al. 1991; Takeda et al. 1988). We investigated the contribution of IL-1β, IL-6, TNF-α, IFN-β and IFN-γ to MDI activity in IV-C-sup. Immature form of IL-1β (proIL-1β) protein was accumulated within cultured chorion cells in response to influenza virus infection, although IL-1β protein was not secreted from the cells (Uchide et al. 2006a). Considerable amounts of IL-6, TNF-α and IFN-β proteins and a trace amount of IFN-γ protein were secreted from the virus-infected chorion cells prior to undergoing apoptosis (Uchide et al. 2002b, 2006a). The induction of both adhesion and NBT reduction abilities was well correlated with the increase of IL-6 protein concentrations in IV-C-sup (Uchide et al. 2006b). It is known that IL-6 receptor α-chain (gp80) binds to IL-6 (Kishimoto et al. 1992), whereas IL-6 receptor β-chain (gp130) itself does not bind to IL-6, but associates with the α-chain/IL-6 complex and is responsible for signal transduction (Taga et al. 1989). The addition of respective antibodies against IL-6 and its receptor subunits, gp80 and gp130, inhibited the induction of adhesion and NBT reduction abilities by IV-C-sup (Uchide et al. 2006b). The combination of these antibodies suppressed >60% of NBT reduction induced by IV-C-sup (our unpublished data). Moreover, the addition of respective antibodies against TNF-α and IFN-β also inhibited. Although the addition of antibody against IFN-γ inhibited the induction of NBT reduction ability by recombinant human (rh) IFN-γ, it did not inhibit the inducible effect of IV-C-sup on NBT reduction. In addition, both superoxide production and adhesion abilities were reconstituted with recombinant cytokines (e.g. rhIL-6, rhTNF-α and rhIFN-β). It is reported that IL-6, TNF-α and IFN-β molecules are heat-stable at 56 °C for 30 min, but IFN-γ molecule is labile (Roberts and Vasil, 1982; Uchide et al. 2006a; Wiedbrauk and Burleson, 1986). On the basis of these results, our studies suggest that MDI activity is predominantly influenced by IL-6 molecule in culture supernatants, and partly by TNF-α and IFN-β, but not IFN-γ, molecules.

MDI activity is also detected in the supernatants of amniochorion tissue homogenate. Influenza virus infection promoted apoptotic cellular degradation in isolated amniochorion tissues in organ cultures and stimulated the secretion of MDI activity, and IL-6 and TNF-α proteins from the tissues (Uchide et al. 2006a, 2006b). It is possible that chorion cells contribute to the production of MDI factors containing IL-6 and TNF-α by amniochorion tissues in response to influenza virus infection. Monocytes/macrophages are normally present in the maternal decidua tissues in large numbers but limited numbers in the fetal chorion and amnion tissues (Vince and Johnson, 2000). Interaction of peripheral blood mononuclear cells with vessel wall involves initial tethering, rolling and firm adhesion to the endothelium, followed by their extravasation to the subendothelial space. Following transendothelial migration, monocytes come into close proximity with subendothelial matrix macromolecules. It has been demonstrated that monocytes are differentiated to macrophages by the contact with extracellular matrix proteins, such as type I and IV collagen and fibronectin (Jacob et al. 2002). This suggests that macrophages are recruited into normal tissues by the contact of monocytes with matrix proteins in physiological conditions. In contrast, various infectious agents induce the expression of pro-inflammatory cytokine genes. It has been demonstrated that pro-inflammatory cytokines, such as IL-1α and TNF-α, stimulate the adhesion and transendotherial migration of monocytes (Kindle et al. 2006), and that monocytes are differentiated to mature macrophages by MDI factors containing IL-6, TNF-α and IFN-β without the contact with matrix protein. Accordingly, in pathological conditions, it is likely that many matured macrophages are recruited into the inflamed tissues infected with influenza virus by MDI factors secreted from the host cells independent on the contact with extracellular matrix proteins.

### Gene regulation of MDI factors

Pyrrolidine dithiocarbamate (PDTC) and 1-β-D-ribofuranosyl-1, 2, 4-triazole-3-carboxamide (ribavirin) are shown to inhibit the replication and transcription of influenza virus gene (Uchide et al. 2002d, 2002e; Uchide and Ohyama, 2003). Both reagents also inhibited the induction of IL-6 and TNF-α mRNA expression in chorion cells after the virus infection and the secretion of IL-6 and TNF-α proteins from the cells (Uchide et al. 2007a, our unpublished data). These results suggest that the synthesis of viral macromolecules is prerequisite for the induction of the expression of pro-inflammatory cytokine genes, such as IL-6 and TNF-α, in chorion cells after influenza virus infection. Since nordihydroguaiaretic acid (NDGA) is shown to inhibit influenza virus proliferation in chorion cells (Uchide et al. 2005b), it is predicted that NDGA can also inhibit the induction of pro-inflammatory cytokine gene expression as well as PDTC and ribavirin.

The transcription of IL-6, TNF-α and IFN-β genes is activated by transcription nuclear factor (NF)-κB (Baeuerle and Henkel, 1994). Since the expression of influenza virus genes, such as hemagglutinin, matrix protein and nucleoprotein, activates the NF-κB-dependent transcription of luciferase gene (Pahl and Baeuerle, 1995; Flory et al. 2000), it is possible that the transcription of IL-6, TNF-α and IFN-β genes is activated in chorion cells in response to the synthesis of influenza virus macromolecules.

Inhibitors of p38 mitogen-activated protein (MAP) kinase, SB203580 and SB202190, inhibited TNF-α protein secretion from chorion cells after influenza virus infection but not TNF-α mRNA accumulation and viral gene replication and transcription in the cells (Uchide et al. 2007a). Therefore, the study suggests that a common p38 MAP kinase pathway is involved in the process of pro-inflammatory cytokine gene expression in the virus-infected chorion cells at a post-transcriptional level.

It has been demonstrated that the human fetal membrane tissues in organ culture produce not only pro-inflammatory cytokines but also anti-inflammatory cytokines, such as IL-10 and transforming growth factor-β. Furthermore, IL-10 showed down-regulation of mRNA expression and protein production of pro-inflammatory cytokines, such as IL-1β, IL-8 and TNF-α, in the fetal membrane tissues stimulated with bacterial toxin lipopolysaccharide (Uchide et al. 2005a). It seems that the balance between pro- and anti-inflammatory cytokine productions is important to control inflammation of the fetal membranes. Although the mRNA expression of IL-1β, IL-6, TNF-α, IFN-β, IFN-γ and granulocyte monocyte colony-stimulating factor in chorion cells was induced by influenza virus infection (Uchide et al. 2002b), the expression of IL-10 mRNA was not (our unpublished data). Therefore, it is likely the production of IL-6, TNF-α and IFN-β by chorion cells in response to influenza virus infection plays a beneficial role in the innate immune mechanisms against the virus infection through the induction of monocyte differentiation to mature macrophages. Therefore, pregnancy-associated complications could occur when the induction of pro-inflammatory cytokine production by influenza virus infection overwhelms a capacity of anti-inflammatory cytokines in the fetal membranes.

## Conclusions

**Figure 1:**
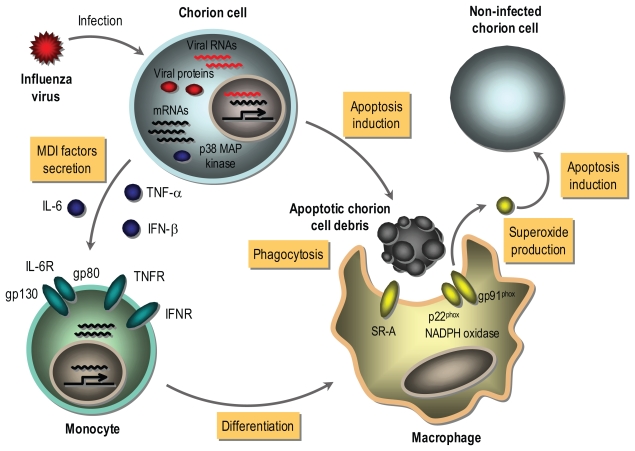
A possible tissue injury model of fetal membranes during intrauterine influenza virus infection MDI factors containing IL-6, TNF-α and IFN-β are secreted from chorion cells in response to the synthesis of influenza virus macromolecules prior to apoptotic cell death. The MDI factors bind to their receptors on maternal monocytes in the decidua tissue, resulting in the expression of SR-A and gp91^phox^ genes. The matured macrophages phagocytose apoptotic chorion cell debris resulting from the virus infection. It is possible that an abrupt increase in superoxide production by phagocyte NADPH oxidase occurs during phagocytosis, and superoxide induces apoptosis in non-infected chorion cells. MDI factors-relating these pathways represent part of mechanisms of pregnancy-associated complications during intrauterine influenza virus infection.

As illustrated in Figure 1, it is possible that the gene transcription of MDI factors containing IL-6, TNF-α and IFN-β is activated in chorion cells responding to the synthesis of influenza virus macromolecules (i.e. viral proteins and RNAs) prior to undergoing apoptosis. MDI factors are secreted from the virus-infected chorion cells depending on the induction of mRNA expression, and a common p38 MAP kinase pathway is involved in the process of gene expression of MDI factors at a post-transcriptional level. Secreted MDI factors bind to their receptors on monocytes in the decidua tissue. After that, monocytes are differentiated to well-matured macrophages capable of phagocytosing and producing superoxide through NADPH oxidase as a result of the induction of SR-A and gp91^phox^ gene expression by MDI factors. The matured macrophages phagocytose apoptotic chorion cell debris resulting from the virus infection. NADPH oxidase present in phagocytes is activated during phagocytosis, resulting in an abrupt increase of superoxide production known as oxidative burst (Minakami and Sumimoto, 2006). The production of superoxide by phagocyte NADPH oxidase is necessary for remodeling tissues damaged by infectious agents (Cohen et al. 1981). However, it is known that an excessive production of superoxide by phagocyte NADPH oxidase is implicated in the pathogenesis of influenza virus in the infected organs (Snelgrove et al. 2006). Apoptosis of chorionic trophoblast cells in the amniochorion tissues obtained at the end of pregnancy was progressed by the in vitro incubation, which was suppressed by the addition of antioxidative reagents [PDTC, NDGA, *N*-acetyl-L-cysteine and 6-hydroxyl-2, 5, 7, 8-tetramethylchroman-2-carboxylic acid (Trolox), a water-soluble analogue of vitamin E], general and selective cyclooxygenase (Cox)-2 inhibitors (indomethacin and nimesride, respectively) and inducible nitric oxide synthase (iNOS) inhibitor (2-amino-5, 6-dihydro-6-methyl-4H-1, 3-thiazine) to the medium (Ohyama et al. 2001; Yuan et al. 2006). The expression levels of Cox-2 and iNOS mRNAs as well as proteins were increased in the isolated chorion tissues during the in vitro incubation (Yuan et al. 2006), resulting in the production of reactive oxygen species, such as superoxide and nitric oxide. Furthermore, apoptosis was induced in cultured chorion, but not amnion, cells by the treatment with a nitric oxide donor reagent, sodium nitroprusside (Yuan et al. 2006). It has been known that peroxynitrite, a strong oxidant, is formed when superoxide and nitric oxide are produced at near equimolar ratios (Virag et al. 2003), and that Trolox inhibits peroxynitrite-mediated apoptosis in rat thymocytes (Salgo and Pryor, 1996). These results suggest that the induction of apoptosis in the chorionic trophoblast cells is mediated through peroxynitrite resulting from the induction of Cox-2 and iNOS gene expression. Conceivably, superoxide produced by macrophages phagocytosing apoptotic chorion cell debris resulting from influenza virus infection may injure the fetal membranes through inducing apoptosis in non-infected chorion cells in vivo situation. Consequently, MDI factors derived from influenza virus-infected chorion cells undergoing apoptosis play a possible pathological role in pregnancy through these pathways. Since PDTC and NDGA exhibit not only antiviral activity but also superoxide-scavenging activity (Floriano-Sanchez et al. 2006; Shi et al. 2000), they are candidates for a drug of choice for anti-influenza treatment as multifunctional agents with antiviral and antioxidant activities (Uchide and Toyoda, 2007c).
